# The Role and Impact of Radio Listening Practices in Older Adults’ Everyday Lives

**DOI:** 10.3389/fpsyg.2020.603446

**Published:** 2020-12-18

**Authors:** Amanda E. Krause

**Affiliations:** ^1^Department of Psychology, James Cook University, Townsville, QLD, Australia; ^2^Melbourne Conservatorium of Music, The University of Melbourne, Melbourne, VIC, Australia

**Keywords:** radio, everyday listening, older age, well-being, quality of life, companionship

## Abstract

Previous research has indicated older adults value listening to music as a leisure activity. Yet, recent research into listening practices broadly has often focused on younger adults and the use of newer, digital listening technologies. Nonetheless, the radio, which is familiar to older people who grew up with it at the forefront of family life, is important to consider with regard to listening practices and the potential associated well-being benefits. This research investigated older adults’ everyday radio listening practices, in order to begin to understand how the radio fits into their daily lives and how it might influence their sense of well-being. Twenty-five Australian residents (aged 66–87; 56% female, 44% male) participated in semi-structured, one-to-one interviews. The results of a qualitative thematic analysis revealed themes concerning listening preferences, listening routines, access, and motivations/outcomes. While personal preferences (concerning content, stations, and presenters) were diverse, individuals clearly communicated these as well as their established listening routines and habits. Listener motivations varied: some people focused on the enjoyment that listening to the radio creates while some noted benefits to their well-being, such as relaxation, modifying their mood, and feelings of comfort and community. Radio listening practices can be defined in terms of differing engagement styles, as characterized using continua ranging from passive to active, or focused, listening as well as generalized or specific listening. Based on participants’ experiences, a proposed engagement space model links how people engage with the radio to the possible outcomes mentioned. Importantly, benefits to well-being can result from varied engagement styles. The findings presented provide an in-depth understanding of how the radio fits into older adults’ everyday life, with implications for considering how the radio might be used as a widely accessed, low-cost tool for maintaining and enhancing quality of later life.

## Introduction

Radio in Australia has a long-running history, with some of the largest listening audiences for radio anywhere in the world (Meadows and Foxwell, 2011). Figures indicate that approximately 28% of the 5.9 million Australians who listen to community radio are aged 55 years or older, and 29% of the approximately 10.9 million Australians who listen to commercial radio are aged 55 years or older (Community Broadcasting Association of Australia, 2019). People aged 55 years or older typically spend 13 h listening to community radio per week (Community Broadcasting Association of Australia, 2019). These statistics indicate that older adults regularly engage with radio listening. However, they only provide us with demographic considerations of “who” listens, highlighting the need for further research in order to consider people’s motivation for listening to the radio as well as the function of radio listening in people’s lives. Indeed, researchers have previously argued that demographic segmentation of audiences often lacks deeper understanding of consumption motivations and outcomes, unless approached from a psychological standpoint (e.g., Krause and North, 2016).

In Australia, the radio has served as an important medium of communication, providing an ever-present and reliable means of communication that transcends both geographical and social boundaries (Foxwell, 2012; Meadows, 2013; Oliveira, 2013; Watson, 2016). Radio programming provides listeners with music, a source of news and information, as well as opportunities for social exchange via interviews, chat-based and talkback programming (Bednarek, 2014; Ames, 2016; Ewart and Ames, 2016). Radio helps to inform, educate and empower its audience (Watson, 2013). Because (community) radio stations produce great diversity in programming (Foxwell, 2012), radio programs can provide tailored content to specific communities (Meadows and Foxwell, 2011), including tailored content for older adults. For example, Foxwell(2012, p. 170) highlighted how Golden Days Radio (a “senior citizens’ radio station”) broadcasts music from “the 1920s to the 1950s” targeted to older listeners in Melbourne. Moreover, an evaluation of the Silver Memories satellite radio service provided evidence of significant improvements to the quality of life for older adults living in aged care after listening to Silver Memories for 12 months as a part of their regular activities (Travers, 2019). For many older Australians, listening to the radio was at the forefront of everyday family life, preceding TV and the use of newer digital listening technologies. Therefore, the radio may hold a special place for many older Australians – whether for nostalgic value or habitual listening.

Research on community radio suggests that it can enhance well-being (Meadows et al., 2007; Oliveira, 2013; Order, 2017). For instance, older adults gain a sense of purpose and identity by volunteering at radio stations (Order and O’Mahony, 2017). Additionally, radio can promote social capital, community development, and social cohesion (Vuuren, 2002; Milan, 2008; Maina, 2013). In linking people together (Oliveira, 2013) and assisting with social relationships (Vidal, 2019), radio can help to contribute to a sense of community (Maina, 2013). For instance, volunteers experience a sense of belonging to community stations (Vuuren, 2002); and there is evidence that community radio listeners who tune in often become more active in the community (Milan, 2008) and can build networks by attending social events advertised on air (Keough, 2010). There is some additional evidence that radio can provide companionship for individuals who are disconnected and isolated (Ewart, 2011). However, specific consideration of the role of listening to the radio in older adults’ everyday lives is needed.

When considering the role of the radio in older adults’ everyday lives, it is also important to consider the impact of listening on people’s well-being. Recognizing that music makes up a large amount of broadcasted radio content, it is possible to draw on work done on music listening in order to hypothesize how radio listening might feature in everyday life. Music listening is one of the most common leisure activities reported across age, race, gender, and culture groups (Schäfer et al., 2013; Koehler and Neubauer, 2020), and it is firmly embedded into our everyday lives (Krause et al., 2015). Research findings indicate that older adults often listen to music (Laukka, 2007), and do so in order to regulate moods, reminisce, facilitate social connectedness, provide enjoyment, and to express identity (Laukka, 2007; Hallam et al., 2012; North and Hird, 2020). Additional research indicates that older adults can focus on different functions of music listening than younger adults, such that there may be differences in the functions of listening depending on one’s age (Groarke and Hogan, 2016). For instance, Groarke and Hogan (2016) found that older adults prioritized mood regulation and the therapeutic benefits of listening. Moreover, Laukka (2007) found that older adults’ listening strategies were correlated to perceived well-being.

Indeed, a growing body of research provides evidence that the arts – and music in particular – can support well-being in older age (Fraser et al., 2015; Cann, 2017), with numerous therapeutic, health, and well-being benefits identified, including those relating to mood, self-esteem, social, cognitive, physical, and quality of life (MacDonald, 2013; Krause et al., 2018). In the domain of psychosocial well-being, it is acknowledged that music assists individuals in forming bonds, fostering a connection with people and community, and provides opportunities for social interaction (Coffman, 2002, 2008; Adderley et al., 2003; Dingle et al., 2012; Rohwer and Rohwer, 2012; Creech et al., 2013; Schäfer et al., 2013). Additional benefits include mood regulation (Garrido et al., 2016), counteracting feelings of depression, anxiety, and pain (Hays and Minichiello, 2005; Jacob et al., 2009; Costa et al., 2018a,b), producing positive emotions and opportunities for self-expression (Bailey and Davidson, 2005; Lehmberg and Fung, 2010; Livesey et al., 2012; Judd and Pooley, 2014), and bolstering quality of life (Hillman, 2002; Hays, 2005; Clift et al., 2008; Gembris, 2008; Tsugawa, 2009; White, 2016).

Of the research that has been conducted on music listening and well-being in older age, most studies have focused on the benefits for older adults with dementia (e.g., Davison et al., 2016; Griser et al., 2016; Garrido et al., 2018; Murphy et al., 2018; Weise et al., 2018; Kulibert et al., 2019), such that there is little empirical evidence relating to older people’s every day, informal listening behaviors (Krause and Davidson, unpublished). Furthermore, despite the high levels of engagement in music listening across the lifespan, research has often focused on adolescents and young adults (e.g., Albarran et al., 2007; Ferguson et al., 2007; McClung et al., 2007) and has tended to concentrate on newer, digital technologies (e.g., Krause and North, 2016); subsequently, more traditional technologies, such as the radio, have slipped from focus. Consequently, little is known about the role of radio in the everyday listening practices of older adults and how these how listening practices might relate to psychosocial well-being.

## Research Aims

The present study was interested in considering how older Australians’ radio listening practices may influence their psychosocial well-being. The research was approached from a social psychological perspective. Given the predominance of music content in radio broadcasting, the research evidence concerning the associated well-being benefits of music listening offers potential ways that listening to the radio may promote well-being. However, the question remains whether such benefits identified with music may apply to radio listening broadly (given radio listening may involve other types of content, such as sport, news, etc.). Research must specifically consider radio listening broadly in order to understand if what is relevant for music is transferrable or generalizable to radio listening. Therefore, this research aimed to investigate older adults’ everyday radio listening practices, in order to understand the role and impact of radio in older adults’ everyday lives.

The first research question concerned how the radio fits into older adults’ daily routines. This question focused on gathering information as to the what, when, where, why, and how of daily radio listening practices. It was anticipated that people would have varied preferences, which may be related to particular radio content, stations, and presenters, and that these personal preferences likely underpin listening habits. Additionally, listening habits were expected to relate to other pursuits (e.g., work and leisure activities), but no particular hypotheses were made.

The second research question investigated how listening to the radio might influence older adults’ sense of well-being. While the present research was exploratory in nature, it was proposed that results might align with existing findings concerning music listening and well-being. In particular, given people commonly listen to music to regulate their mood (Lonsdale and North, 2011; Schäfer et al., 2013), it was proposed that older adults may listen to the radio to regulate their moods. Additional work on radio involvement (e.g., Meadows and Foxwell, 2011) suggests that listening may also promote feelings of connection and community. It was anticipated that participants might mention additional listening motivations and outcomes that speak to how listening to the radio might impact well-being.

## Materials and Methods

### Sample

A sample of 25 Australian residents (56% female, 44% male) participated in one-to-one interviews. The participants were aged 66–87 (*M* = 74.88, *Mdn* = 74, SD = 6.62), and all resided in the metropolitan/regional Melbourne area. Most of the sample was Australian (22, or 88.00%), with the three remaining individuals reporting their nationality as Australian-American, German, and New Zealander.

Participation in the study was voluntary; however, as remuneration for their time, each participant received a $25 AUD gift card. Recruitment included the use of online tools (including a project webpage and news articles); radio adverts and short interviews; and social media postings.

### Design and Procedure

A qualitative enquiry methodology involving semi-structured, individual interviews was used. A conversational style was used to explore key ideas: the main research questions were pre-determined, but the semi-structured nature allowed for tailored exploration of each participant’s experience (Bhattacharya, 2017). Interviews offer a fruitful method of qualitative data collection when working with older adults (Klein and Parks, 2007; Tkatch et al., 2017), and their use responds to calls for the use of such methods in order to understand experiences of everyday activities as well as aging (e.g., Kelly, 2010; Phoenix, 2018). Interviews were scheduled at a time and location suitable to each individual. Participants were provided with information about the study and consented to participate prior to the interview commencing. Individuals were asked to state their age, gender, postcode, and nationality on the consent form in order to report on the demographic details of the sample.

Key questions elicited their responses about listening practices, including probing what, when, how, and why they listen to the radio (i.e., “Could you tell me about how and why you listen to the radio?”, “What do you like to listen to on the radio?”). Listening routines and motivations were explored (i.e., “Do you have any listening routines?”, “When do you listen – are there any certain times you listen to the radio?”), and an additional lifestyle question was asked to understand how the radio featured in their daily and weekly life (i.e., “How does your week work … how does the radio fit in?”). As such, the data collected was based on participants’ reflections of their own listening practices and experiences.

Ethics approval for this study was obtained from the human ethics committee at The University of Melbourne (Ethics ID: 1749766).

### Data Analysis

All interviews were audio recorded with the consent of the participants. Each interview recording was transcribed verbatim. A thematic analysis, following Braun and Clarke’s (2006) six-step procedure, was used to analyze the interview data. This procedure begins with familiarizing oneself with the data, and progresses to generating codes, identifying and reviewing themes, in order to conclude by labeling and reporting on the themes (using quotes to support each theme). A recursive, reflexive thematic analysis approach was adopted whereby examination of the participants’ responses was flexible, rather than focused on any specific theoretical background. Semantically similar interview responses were identified within the entire dataset, and initial codes were generated to capture the data. Following this, tentatively, broader themes were formulated by clustering related codes. While semantic similarities primarily guided the formulation of these themes, coding of implicit concepts within participant responses were explored and included where relevant. To best represent the data relative to the research questions, higher order themes and sub-themes were developed through refining the themes (allowing for re-naming and/or combining themes). To uphold participant confidentiality, participant quotes appear alongside the participant’s gender and an age range.

## Results

A total of four themes characterize the older adult participants’ radio listening practices and experiences. These themes include: preferences; listening routines; radio access; and motivations and outcomes. Given the nature of the interview questions, the findings address the “six Ws” of information gathering (i.e., who, what, where, when, why, and how). Each theme is considered below, supported by participant quotes.

### Preferences (the “What”)

Participants stated their personal preferences clearly, often clarifying their preferences by also stating what they did not like. These content-based programming preferences (e.g., music, news, talkback) underpinned people’s engagement with the radio, as people also expressed their preferences in terms of stations, as well as particular radio programs and presenters.

With regard to content preferences, many of the sample preferred music programming. As anticipated, interviewees expressed varied personal music preferences. Multiple music genres were referenced across the sample; light classical and pop music were often mentioned. Some participants had wide preferences [e.g., “I have an incredibly diverse taste in music. I will listen to opera and I absolutely love it. I also, at the other end of the scale, love blues and rock music…. Also love jazz and folk music. There’s a lot of country music that I really enjoy too.” (Male, 70–74)], while others had narrow preferences [e.g., P01: “it’s almost entirely opera” (Male, 65–69); P11: “I like classical music only” (Female, 75–79)].

In addition to music, some participants preferred listening to the radio for news and current affairs [“my primary interest is the news and how that’s developed and the sporting channel” (Male, 80–84); “I listen to the ABC news channel at night which gives me the BBC. So I can get the world news.” (Male, 80–84)]. However, participants were divided as to whether or not they enjoyed talkback radio programming [e.g., “I cannot bear to listen to the talkback” (Male, 65–69) versus “in the morning, I quite like talkback” (Female, 75–79)], and very few participants mentioned listening to the radio for sports.

In addition to simply naming preferred local commercial and community radio stations (e.g., ABC, Classic FM, 3MBS, Golden Days Radio), it was apparent that people’s preferences also depended on people’s listening habits. Some participants’ preferred a particular channel [“I just listen to the ABC” (Male, 65–69); “I listen to … FM radio – classical music. …“I don’t flip through the stations. I just turn on the FM and that’s it.” (Female, 75–79)], while others listened to multiple channels. Indeed, some participants could be considered as channel “surfers” – switching the dial in search of what they wanted to hear:

“If there’s any sort of pop or folk music I turn it off straight away. It doesn’t give me any joy.” (Female, 75–79)

”if I don’t like one thing, I’ll go over to another station” (Female, 80–84)

It seems that people’s preferences drive channel-switching behavior; however, in contrast, a few participants were happy to turn on a station and leave it there [e.g., “I don’t go around turning things off” (Male, 80–84); “So long as it’s music I like and it’s on the radio, I’m happy with that.” (Female, 65–69)].

Participants also named particular programs [e.g., “Blues with a feeling. That’s often one I listen to” (Male, 65–69)] and/or presenters of interest [e.g., “I like Jon. I particularly like listening to him” (Female, 65–69); “I love Phillip Adams … This week he’s not on. There’s a woman on instead and it’s not the same.” (Female, 75–79)]. By expressing preferences for presenters (or certain presenter behaviors), it is evident that radio presenters also play an important role for listeners. Presenter style is important [“there are a couple of really great presenters … their knowledge of music and their personalities” (Male, 85–89)], such that people can change their listening practices if they do not like the presenters [“All the radio announcers talk too fast. …I’ll put up with the speed if I’m interested in the content” (Female, 75–79); “I don’t enjoy listening to quite a number of the presenters..maybe they’re building a whole new regime. I don’t know what they’re up to, but I don’t enjoy it.” (Female, 85–89)]. For instance, some individuals mentioned how they have searched for alternate programs to listen to because of changes in program presenters [“I normally listen to the ABC in the morning, but now I don’t like the presenter” (Female, 80–84)].

Lastly, individuals’ preferences were, in part, dictated by what they could access [“sometimes if I’m in the area of Caulfield I listen to … the Jewish channel … because of the transmission capability” (Male, 70–74)]. However, participants also expressed unfulfilled listening desires/preferences. For example, some participants wished that there were more radio plays broadcasted [“I discovered radio plays, and I used to love them. I used to really love them. But then they sort of vanished, and they don’t seem to appear any longer.” (Male, 65–69)].

### Radio Listening Routines (the “When” and the “Where”)

It was apparent that most of the sample listened to the radio most days, although the amount of time spent listening differed. The participants’ listening practices, or routines, can be differentiated using a general-specific continuum. Beyond focusing on or surfing channels (as discussed above), people tended to either seek specific programming or were content with what they happened upon [e.g., “whatever the channel happens to be” (Male, 70–74)]. Listening routines at the “specific” end were driven by both programming preferences and listening at certain times of the day:

Male, 65–69, who prefers opera: “we got those two radio programs on Saturday. Tuesday, there’s the Tim Gaffney in two hours. Wednesday night, they’ve got ‘Wednesday night at the opera’ … There’s another one – Friday night, there’s one at seven o’clock, a half hour of opera music. I always try to get all those programs” Male, 65–69, who listens in the evenings: “at home it would be at an evening. Often before we go to bed, before we go to sleep, we would put on the digital radio …I would have ClassicFM or I would have Jazz or Smooth Hits.”

Indeed, many people’s radio listening practices revolved around sleeping routines. This included routines connected to waking up [“particularly first thing in the morning because that’s my alarm” (Female, 80–84); “I would usually use it when I’m getting up, getting dressed early in the morning.” (Female, 65–69)], as well as going to bed [“so at night when I go to bed I put the radio on and I leave it on all night” (Male, 65–69); “I can’t bear just silence. If I’m trying to go to sleep or I’m tired I’ll have something on.” (Female, 65–69)]. Additionally, listening routines pertained to having trouble sleeping [“instead of reading a book because it disturbs with the light … the radio seemed like the best bet in terms of working out how I can just lie there and relax a bit, …and then go back to sleep.” (Female, 70–74)]. For some, the radio is even left on overnight [”when I turn off the TV at night, I put the radio on 105.9 usually. …and that might go quietly while I’m asleep, then I’ll wake up, there it is.” (Female, 80–84)].

The clearest contrast to specifically listening at certain times was by those who expressed a habit of having the radio on all day:

“I have the radio on, if I’m at home, I have the radio on all day. I go in and out, it’s on – I leave it on.” (Male, 70–74)

“I turn it on as soon as I wake up. If I’m in the car, music’s on. I have four radios and if I change rooms the radio goes on in a new room.” (Female, 65–69)

“It just runs all day. …Anyone that comes in says, isn’t the music lovely. It fills the whole apartment with a feeling of relaxation.” (Female, 85–89)

For some, listening routines were also tied to locations [e.g., “I’m quite often anchored in (the kitchen) and so the radio is always there. …So if I’m working around the kitchen, cooking or whatever, it’s there and convenient” (Female, 80–84)]. Listening to the radio at home was most common, followed by listening in the car [”basically every time I’m in the car…I’d always put the radio on” (Male, 65–69)]. For some listening in the car occurred in addition to listening at home; for others it was the only place [e.g., “I don’t listen during the day except in the car. We don’t have a radio running during the day” (Female, 75–79); “only when I’m in the car driving. I haven’t got time otherwise” (Female, 70–74)].

Additionally, however, some people’s radio listening practices traversed space, by exploiting the portability of radio [“because the radio is portable, I can carry it with me” (Female, 80–84)] or by using multiple devices:

”sometimes if I’m in my study which is at the other end of the house and I go and I leave the radio on in there, and then I’ll go in the kitchen and start doing something else but then I’ll put the radio on in there, because I can’t hear this one unless I have it really loud. So I always put one in every room I’m in.” (Female, 65–69)

It should also be noted that a few participants mentioned having to negotiate their radio listening with partners. Additionally, a few participants stated that they did not have set routines, such that listening to the radio can also be more *ad hoc* [“When I feel like it” (Male, 70–74); “There’s not a time of the day when I would say, oh it’s two o’clock, I’ll sit down and listen to such and such. No, it doesn’t govern my time in that way” (Female, 80-84)].

### Radio Access (the “How”)

Listening to the radio can involve the use of a variety of technology. Most people were using traditional devices (radios, stereo systems); however, some participants were using cable TV, computers, as well as applications on smartphones and tablets. Moreover, for some, the radio was the primary media they listened to [e.g., “it’s really just radio” (Female, 70–74); “mainly radio” (Female, 65–69)]. Others complimented listening to the radio with listening to music in other formats [e.g., “the best thing is to go and hear (music) live, by far in a way the best. And then the radio, television, Foxtel… there’s a lot of CDs over there but I don’t listen to them anymore, YouTube’s taken over”… “you can get everything on YouTube” (Female, 80–84)].

### Motivations and Outcomes (the “Why”)

This theme speaks to both *why* older adults listen to the radio (their motivations and reasons) as well as the potential outcomes, and benefits, of listening to the radio. These were grouped into a number of sub-themes: enjoyment, information, company and comfort, mood regulation, reminiscence, creating an atmosphere, and to pass the time (as accompaniment to other activities).

#### Enjoyment and Information

Radio listening provides enjoyment [e.g., “I just enjoy it … I’m not really trying to get anything out of it except just the enjoyment” (Male, 65–69)] as well as information [e.g.,“seeing what’s happening … find out what’s happening and also the weather forecast and so on.” (Male, 85–89)]. Beyond getting the news and “being aware of what’s going on” (Male, 65–69), participants also spoke about how what they hear helps them to “develop opinions” (Male, 80–84) [and “for me it’s sort of keeping a little bit more in touch, and also what people’s views are, and then make an assessment about [what] I think” (Female, 65–69)].

#### Company and Comfort

Many participants found that listening to the radio provided company:

“mostly I listen to it for background music, and, when I’m by myself, company” (Female, 75–79)

”I think when you’re stuck around the house, it’s just a great - not going to say friend, it’s good company, you know” … “something I can listen to, instead of hearing my footsteps around the house” (Female, 80–84)

”I put it on sometimes for the dog so she has company when I go out” (Female, 75–79)

Participants also spoke of how, by providing company, the radio can provide comfort and warmth:

”It’s warmth, a sense of warmth … in the background, so it’s not that there’s just nothing happening. … And you kind of don’t feel alone. yeah, don’t feel alone” (Male, 65–69)

For some, an intimacy came with listening to the radio:

“I love the intimacy of radio. So, I would use radio far more than television. …The intimacy of radio is what I love. …I just like its companionable nature that works for me with radio” (Female, 65–69)

”listening to the radio when you’re in bed, it’s quite intimate I guess in a way, and it’s quite close because the radio is quite close to you and you can hear people talking and whatever, and it’s quite pleasant.” (Female, 65–69)

#### Mood Regulation and Relaxation

Some participants consciously sought out listening to the radio to shift their mood and feel better. In nearly every case, these individuals sought out music programming for this goal:

“If I’m feeling really down, and I know that I am, I will try and put music on that I know will bring me out of that” (Female, 80–84)

“I would have the odd day when I’m perhaps feeling a little grumpy or a little not quite the way I want to feel and I might put a bit of jazz on and start tapping and moving with the jazz. …It’s often a good mood changer if you need it.” (Male, 65–69)

In addition to trying to feel better, people also listened to the radio to help them relax:

”It just takes me to another sphere. It tends to just give me that feeling of great peace and enjoyment.” (Female, 85–89)

“I want soothing type of music and just to wind down and to sometimes drift off to sleep with.” (Male, 65–69)

#### Creating an Atmosphere

While not mentioned by many participants, another sub-theme addressed using the radio for creating an atmosphere:

”I’m not used to having a silent atmosphere, I’d rather have some sounds.” (Female, 80–84)

“it sets up like a beautiful atmosphere and with that atmosphere you can then be more in harmony with your surroundings.” (Male, 85–89)

#### Reminiscence

A few participants acknowledged that the “radio brings back that memory or that feeling” (Male, 65–69). In other words, that what people hear on the radio can induce nostalgia [“It brings back very vivid memories of certain things” (Female, 89–84)], and some listened to purposefully reminisce:

“I’m a bit of a thinker, a bit of a reminiscer. So, I would listen to a lot of these things and I’d reminisce about aspects of my life previously – years ago, decades ago – that involved these songs and they’d bring back good memories. If something comes on that doesn’t bring up a good memory, I’d switch it off.” (Male, 65–69)

#### Passing Time

For many, listening to the radio helped pass the time. People often put the radio on in the background when doing other things. In this way, the radio was used as an accompaniment to other activities.

”it passes the time” – “makes it more interesting. When you’ve been cooking for 60 years or whatever… it passes the time to cook and listen” (Female, 70–74)

”so when I’m vacuuming, while I’m washing the floors … I’ll turn it on and listen … it really takes my mind off the work that I’m doing” (Male, 70–74)

“when I’m painting I love it. To me one of the best things in my life is painting and listening” (Female, 75–79)

#### Content Type, Motivations, and Outcomes

It may be that different types of radio content may be associated with different listening motivations or outcomes. As one participant stated, “To the radio, I listen mainly for information on politics … I keep listening to the music for the enjoyment of it” (Male, 70–75). Based on the participants’ comments, it seems that spoken content (news, interviews, etc.) provided information, whereas music supported reminiscing, regulating moods, relaxing, and setting the atmosphere. In contrast, any preferred radio content could assist listeners looking for enjoyment or entertainment, seeking to pass the time, or have background sound as they do other things like household chores. Additionally, it seems the radio, broadly, can provide companionship.

#### Passive and Active Listening

It is also interesting to consider people’s engagement with the radio in terms of another listening continuum – that of passive-to-active (or focused) listening. In fact, it may be more appropriate to label the ends of this continuum with regard to whether the radio listening is the primary or secondary activity. There was evidence of people’s listening styles falling at the passive extreme [”to me music is a background to something else that you do. To sit there just listening, I don’t find stimulating. I need to do other things, so I’ll read and I’ll listen to music, or I drive and I listen to music” (Female, 70–74)] as well as the active extreme [“If the radio is on I have to be actively listening otherwise it’s not on. …I hate it as background noise. …I want to hear it or have silence.” (Female, 75–79)]. Largely though, participants’ listening tended to fall somewhere in between those extremes [”I don’t often sit down and deliberately listen for half an hour. … It’s there all the time, and so, you know, I’ll prick up my ears at certain things and other things I can ignore.” (Female, 80–84)].

Further, for some their listening style depends on the type of content (“I listen to the people talking on 612, quite actively listen. I don’t actively listen to music, that’s just in the background.” (Female, 75–79)]. For instance, one participant (Female, 65–69) contrasted their preference for music to “be more in the background because I’m doing something or talking or whatever” with actively listening to talkback radio (“I listen. It doesn’t mean I remember absolutely everything, but I do listen.”).

### Radio Engagement Continuum Model

**FIGURE 1:**
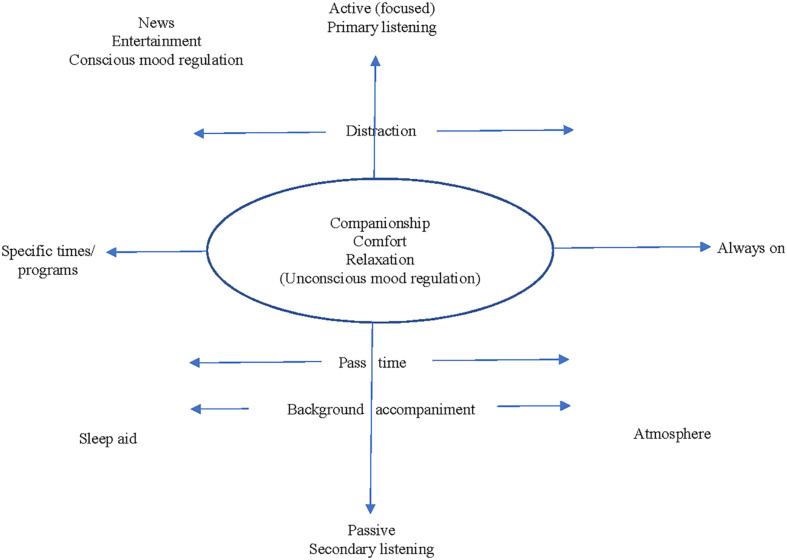
The radio engagement continuum model.

Based on the present findings, the radio engagement continuum model (see Figure 1) uses the passive-active (secondary–primary) continuum and the general-specific continuum (in terms of listening at specific times versus having the radio on constantly) as axes to create a general engagement space model. By plotting these continua, it is possible to see how the ways participants’ engaged with the radio could produce the outcomes mentioned. For instance, it is easy to see how having the radio on playing music on, though purposely in the background all day, can create a pleasant atmosphere. Yet while still considered passive, turning the radio on at a certain time can result in a sleep aid. Many listened to the news at specific times, and actively attended to this content. Not all of the motivations or outcomes sit in a single quadrant, however. For instance, using the radio to pass the time or to provide background sound while doing other activities is passive, though could be placed anywhere along the specific-always on continuum. Additionally, some outcomes cannot be plotted along these two axes. Rather, outcomes, including companionship, comfort, relaxation, and unconscious mood regulation could result from any type of radio engagement, and, thus, they can lie anywhere in the space model.

Indeed, during the interview, participants were asked to consider how they might define their relationship with the radio. Based on reading previous literature and anecdotal stories, contrasting examples were provided as cues: that the radio might provide background sound or offer companionship for listeners. Though having the radio on in the background can suggest a passive (secondary) style of listening, people’s responses made it clear that “companionship” is not necessarily a polar opposite to “background sound.” In other words, companionship transcends both axes, and, as superimposed, could lie in any quadrant.

“It’s both. A bit of background, and sometimes sort of [a companion].” (Female, 65–69)

“Background … there would be companion thrown into that because, as I said, it’s another human voice” (Female, 80–84)

“Companion… even if I’m reading a book, I’ll have music on…[the radio is] always there, I wouldn’t turn it off to go and do something else.” (Female, 65–69)

The perception of the radio’s function, of course, can also vary – both across and throughout listening episodes [“possibly depending on where I am at on that day – sometimes it most definitely is background” (Female, 85–89)]. Thus, any type of listening engagement could result in the listeners perceiving comfort and companionship (noting that the same is true, then, for relaxation and unconscious mood regulation).

## Discussion

The present study examined the role of radio in the everyday lives of older Australians and considered how radio listening may be related to psychosocial well-being. With regard to the first research question, the findings indicate that listening to the radio was well integrated in many of the older adult participants’ daily lives. Nearly all of the participants listened at home, and listening in the car was also very common. The sample reported using a wide variety of technology to access the radio. For some this included using digital technology, countering the long-standing discourse that older people do not use, or cannot use technology (Vines et al., 2015).

Participants largely preferred listening to the radio for news/information and music; and many participants had daily listening routines. For some individuals, these routines included listening to specific programming or at specific times; for others, the radio tended to be always on, accompanying them throughout the day. Interpreting the participants’ comments resulted in using two continua to describe how people engage with the radio. Firstly, listening engagement appears to range from being a passive to active, focused activity (in other words that the act of listening might be a secondary activity to something else or instead draw someone’s attention as the primary activity). Secondly, there appears to be a specific-general continuum with regard to preferred content. Most notably, beyond surfing the dial versus staying on a particular channel, people tend to either seek out specific programming/listen at specific times or have a habit of always having the radio on. Using these two continua as axes, the radio engagement continuum model (Figure 1) can be interpreted as a general model of older adults’ everyday engagement with the radio with respect to both listening motivations and listening outcomes.

Given that radio broadcasting aims include entertaining and informing the listening audience (e.g., Towers, 1987; McClung et al., 2007; Watson, 2013), it is not surprising that participants mentioned that they gained information and experienced enjoyment from their listening. However, in addition, participants’ motivations for engaging with the radio included company and comfort, mood regulation, relaxation, reminiscence, creating an atmosphere, and passing time. These motivations can simultaneously be labeled as outcomes or benefits of listening to the radio. Thus, in considering the second research question, which concerned how listening to the radio might influence older adults’ sense of well-being, it is particularly relevant to consider these themes in more depth.

Firstly, all of these outcomes can be interpreted as positive in nature and as evidence of how listening to the radio can have positive benefits for well-being in older age. As anticipated, these themes mirror those established well-being benefits of listening to music (e.g., Krause et al., 2018), suggesting that key findings from research on music listening and well-being might also be applied to radio. Firstly, it is important to note the fact that some people were aware that their listening could provide companionship and help regulate their mood. Secondly, as seen in the radio engagement continuum model (Figure 1), these particular outcomes were possible from any/all radio engagement styles. Consequently, it is worth interrogating the mood regulation and company and comfort (companionship) themes further.

The participants who spoke of listening to the radio to regulate their moods specifically mentioned listening to music. This is not surprising given that mood regulation is one of the most common reasons people listen to music (Lonsdale and North, 2011; Schäfer et al., 2013). However, it is interesting to consider, as previous research has shown greater positive shifts in mood being associated with having more control over listening in everyday life (e.g., Krause et al., 2015). Selecting a particular album or using a personal playlist, for example, affords listeners the opportunity to pinpoint their listening. While a listener can select a radio station, which narrows the genre, they are not choosing the particular music. Yet, the present study indicates that older adults do shift their mood via radio (music) listening. Perhaps, as one participant commented, this difference in knowing what will be played has positive consequences – “it’s not quite the same [as if I put a record on], because you don’t know what’s coming on the radio and so you’ve got that response to something that’s immediate and you don’t pre-consider what it’s going to be like” (Male, 65–69).

However, it is important to note that listeners may not be consciously aware that their listening may influence their moods. Thus, listeners could still receive this benefit to their well-being, even if not aware [”I’m not conscious of putting something on, but it could be, particularly in this last year, I’ve probably put it on more as a prop. Something to, sort of, help you get through, because there were times when it was a bit difficult.” (Female, 80–84)]. Additionally, it should be recognized that listening could have a negative impact on people’s moods. Previous research exploring healthy-unhealthy uses of music with adolescents, suggests that the power of listening as a coping mechanism be carefully considered (e.g., McFerran and Saarikallio, 2014; Saarikallio et al., 2015). This extends to reminiscing as a listening outcome, as what is heard may not always positive. Again, there is growing evidence of using music to assist with memory and reminiscence in older age (e.g., Istvandity, 2017), though explicit consideration of the radio is warranted.

The present findings suggest that listening to the radio can also provide companionship and create a sense of community, well-being benefits often associated with listening to music (see Krause et al., 2018 for review). Indeed, recent research has demonstrated how listening to music can act as a social surrogate (Schäfer and Eerola, 2020; Schäfer et al., 2020). However, this benefit was not *only* prescribed to listening to music programming on the radio. As one participant stated, part of the reason some people listen to the radio “is because you hear people talking” (Male, 65–69) [and “because you also have the presenters, you have another human voice speaking” (Female, 80–84)]. For another participant (Female, 70–74), the feeling of companionship is created, in part, “especially with the talkback” element of radio – perhaps because of listening to others with similar interests and/or from the local area (Foxwell, 2012). Thus, it is important to consider the role that radio presenters have in creating this well-being benefit. While radio presenters impact people’s continued radio engagement (Stiernstedt, 2014; Vision critical, 2018), the present findings suggest that listeners establish bonds with the presenters they hear on the radio, which can positively influence their perceived well-being. It falls on future research to consider presenter behaviors that are not only well-liked by listeners but that might additionally support feelings of comfort, companionship, and community. Additionally, it would be of great benefit to examine the ways in which presenters and audiences engage and interact when considering how the radio can support well-being.

Of course, not everyone in the sample considered the radio to be a companion. However, this does not diminish the radio’s potential to offer listeners companionship.

”I suppose if I was on my own, it would be companionship. Probably [it is] in the background, as it is now.” (Female, 85–89)

Comments such as that speak to how radio can assist those who feel lonely and isolated by providing opportunities to feel a part of a community (e.g., Ewart, 2011; Foxwell, 2012). However, listeners need not be alone to find company by listening:

”After being sort of housebound for a year, I can really understand how important radio is, just as a company – as keeping you company. I mean I wasn’t always on my own, in fact I was hardly ever on my own. But it just gives you something. It’s a warmth. It just makes so much difference to the day, I think.” (Female, 80–84)

Previous researchers have noted how the radio can play a role in health promotion (e.g., Forde et al., 2009; Ewart, 2011). In other words, the radio can be used to educate and distribute health information to listeners. Thus, it is also interesting to consider how information about radio engagement for well-being benefit might be broadcasted on-air to older adult listeners. Because radio programs, especially those from community stations, can provide tailored content to specific communities (Meadows and Foxwell, 2011), it is possible that programming could be developed that would promote feelings of comfort and community as well as the other mentioned benefits. Designing such programming efforts may be particularly well suited to community stations already targeting older adults. Explicitly discussing how radio engagement may influence well-being would provide older adults with added support in engaging in healthy listening habits.

The present study is not without its limitations; however, these limitations point to interesting lines of future research enquiry. For instance, the study is small in scale and draws on people living in a metropolitan location, such that further work is needed to consider people living in regional and remote locations. Additionally, it did not overtly measure the participants’ perceived well-being or include the perspectives of older adults who do not listen to radio. Future research using quantitative measures of well-being as well as methodologies that utilize real-time data collection (e.g., experience sampling) could further our understanding of listening choices and changes and could assist in further developing the proposed engagement model. Moreover, the present study did not exclusively focus on (or compare) the participants’ engagement with commercial, community, and public radio. However, a few participants mentioned listening routines where these different types of radio were intertwined (e.g., switching from commercial to community stations when presenters changed, and seeking specialist music programming available on community stations – in line with Meadows et al., 2007). Lastly, the focus of the present study was on a more traditional notion of radio broadcasting, which excluded any real focus on podcasts (although some of the participants did include podcasting in their daily routines). Given some of the sample used multiple technologies to access and listen to the radio, future research is still needed to continue to question what is classified as radio (Ariana Moscote Freire, 2007), to specifically consider podcast listening, and to situate engaging with the radio amongst other daily leisure pursuits that may positively influence well-being. Such research will contextualize everyday listening choices and demonstrate the role of situational factors to better understand their role in promoting well-being.

Collectively, the present findings have implications for how the radio might be used as a widely accessed, low-cost tool for maintaining and enhancing older adults’ quality of life. Given Australia boasts the fourth highest life expectancy in the world (WHO, 2016); and, by 2057, close to one in four of the population will be over 65 (Australian Institute of Health and Welfare, 2017), it is important to consider what role the radio might play alongside other non-pharmacological, arts-based approaches to support well-being in older age. As one participant stated, “it’s wonderful that all this stuff is on the radio for nothing” (Male, 65–69).

## Data Availability Statement

The datasets presented in this article are not readily available because the ethics approval did not permit sharing. Requests to access the datasets should be directed to AK, amanda.krause1@jcu.edu.a.

## Ethics Statement

The studies involving human participants were reviewed and approved by The University of Melbourne’s Human Research Ethics Committee. The patients/participants provided their written informed consent to participate in this study.

## Author Contributions

AK conducted the research and developed the manuscript to report the findings.

## Conflict of Interest

The author declares that the research was conducted in the absence of any commercial or financial relationships that could be construed as a potential conflict of interest.
